# Safety of oral ivermectin during pregnancy: a systematic review and meta-analysis

**DOI:** 10.1016/S2214-109X(19)30453-X

**Published:** 2020-01-01

**Authors:** Patricia Nicolas, Marta F Maia, Quique Bassat, Kevin C Kobylinski, Wuelton Monteiro, N Regina Rabinovich, Clara Menéndez, Azucena Bardají, Carlos Chaccour

**Affiliations:** ISGlobal, Hospital Clínic− Universitat de Barcelona, Barcelona, Spain; Department of Biosciences, KEMRI Wellcome Trust Research Programme, Kilifi, Kenya; ISGlobal, Hospital Clínic− Universitat de Barcelona, Barcelona, Spain; Armed Forces Research Institute of Medical Sciences, Bangkok, Thailand; Fundação de Medicina Tropical Dr Heitor Vieira Dourado, Manaus, Amazonas, Brazil; ISGlobal, Hospital Clínic− Universitat de Barcelona, Barcelona, Spain; ISGlobal, Hospital Clínic− Universitat de Barcelona, Barcelona, Spain; ISGlobal, Hospital Clínic− Universitat de Barcelona, Barcelona, Spain; ISGlobal, Hospital Clínic− Universitat de Barcelona, Barcelona, Spain

## Abstract

**Background:**

About 3·7 billion doses of ivermectin have been distributed in mass drug administration (MDA) campaigns globally over the past 30 years. At 10−100 times higher than current human doses, ivermectin is a known teratogen in mammals. During these campaigns with recommended doses, pregnant women might be inadvertently exposed. We therefore aimed to evaluate the existing evidence for serious and non-serious adverse events after ivermectin exposure in pregnant women.

**Methods:**

For this systematic review and meta-analysis, we searched relevant databases and trial registry platforms on July 15, 2018, for randomised controlled trials (RCTs) and observational studies that reported adverse events in pregnant women. We did not use language or date restrictions. Outcomes of interest were spontaneous abortions, stillbirths, congenital anomalies, and neonatal death (serious adverse events), as well as maternal morbidity, preterm births, and low birthweight (adverse events). The risk of bias was assessed using the Newcastle-Ottawa Scale for observational studies and the Cochrane Risk of Bias Tool for RCTs. We did the meta-analysis of observational studies and RCTs separately. The quality of evidence was assessed using the GRADE approach. The study protocol is registered with PROSPERO, protocol CRD42016046914.

**Findings:**

We identified 147 records, of which only five observational studies and one RCT were included for quantitative analysis; these studies were published between 1990 and 2008, and were done in six African countries. 893 women with 899 pregancy outcomes were included, of whom 496 pregnant women (500 pregnancy outcomes) received ivermectin inadvertently during MDA campaigns in the observational studies and 397 pregnant women (399 pregnancy outcomes) purposely received ivermectin as part of the open-label RCT. No study reported neonatal deaths, maternal morbidity, preterm births, or low birthweight. It is unclear whether exposure to ivermectin during pregnancy increases the risk of spontaneous abortions and stillbirths (odds ratio [OR] 1·15 [95% CI 0·75−1·78] with very low certainty of evidence for the four observational studies and 0·62 [0·18−2·14] with very low certainty of evidence for the RCT) or congenital anomalies (OR 1·69 [95% CI 0·83−3·41] with very low certainty of evidence for the five observational studies and 1·10 [0·07−17·65] with very low certainty of evidence for the RCT).

**Interpretation:**

There is insufficient evidence to conclude on the safety profile of ivermectin during pregnancy. Treatment campaigns should focus additional efforts on preventing inadvertent treatment of pregnant women.

**Funding:**

Unitaid.

## Introduction

Ivermectin is a widely used antiparasitic drug.^1–3^ Since 1987, more than 3·7 billion treatments have been donated by Merck through the Mectizan Donation Programme with the goal of eliminating onchocerciasis. In 1998, this donation programme was expanded to include lymphatic filariasis.^4,5^ The global demand for ivermectin is expected to remain high because of its licensure for use against *Strongyloides*, scabies,^3,6^ the potential to eliminate lymphatic filariasis when given as part of a three-drug combination with albendazole and diethylcarbamazine,^7^ combined regimens for soil-transmitted helminths, and its potential role as an endectocide to reduce malaria transmission by killing malaria vectors.^8^

Before moving to the narrative description required by the 2015 labelling rule,^9^ the US Food and Drug Administration (FDA) had previously classified ivermectin as pregnancy category C—ie, “Animal reproduction studies have shown an adverse effect on the foetus and there are no adequate and well-controlled studies in humans, but potential benefits may warrant use of the drug in pregnant women despite potential risks”.^1^ This classification is based on studies done in mice, rats, and rabbits during the original New Drug Application in the 1990s by Merck (appendix p 2).^10^ These studies showed adverse pregnancy outcomes at cumulative doses that are high enough to produce signs of maternal toxicity in animals, ranging between 20 and 600 times the human Mectizan single-dose target of 0·15−0·20 mg/kg. However, later evidence showed that the mouse strain (CF-1) used in the initial acute and developmental ivermectin toxicity studies was inappropriate, as it was later shown that CF-1 mice have deficient P-glycoprotein expression, which is an efflux pump key to preventing ivermectin toxicity.^11^

During ivermectin mass drug administrations (MDAs) for onchocerciasis and lymphatic filariasis, visibly and self-reported pregnant women are excluded from treatment without requiring pregnancy testing.^12^ This omission of testing leads to an unknown number of women at risk of inadvertent exposure to ivermectin early in pregnancy, which could be as high as 50% of women in their first trimester.^13^ In highly endemic onchocerciasis areas, where risk of eyesight loss is high, Mectizan campaigns have included pregnant women at the discretion of the programme.^12^ Research done in the early days of ivermectin MDA for onchocerciasis^14^ showed that simple questioning was the most efficient method to detect pregnancy in this context, although this point might require validation against newer, more sensitive tests. The programme’s decision was based on the large clinical experience of the campaigns, in which inadvertent use in hundreds of pregnant women had no apparent harmful effect. The decision was supported by evidence that P-glycoprotein in the placenta prevents avermectins (the drug family to which ivermectin belongs) from penetrating the placenta.^11,12^ P-glycoprotein also minimises ivermectin-induced neurotoxicity in mammals by active efflux of the drug at the blood−brain barrier, thus preventing ivermectin entry into the CNS.^15^ However, rat and human placental P-glycoprotein expression during gestation differ; in humans, placental P-glycoprotein expression wanes during gestation,^16,17^ whereas it increases in rats.^18^ In general, human blood−brain barrier development begins earlier in gestation and proceeds faster compared with rodents,^19^ with human blood−brain barrier P-glycoprotein detectable as early as 8 weeks of gestation.^20^ Indeed, in humans, the expression of blood−brain barrier P-glycoprotein reaches far higher concentrations during gestation when compared with mice or rats.^21–23^

Weighing the risks and benefits of ivermectin use in pregnancy is imperative for informed public health policy (eg, MDA campaigns), as well as for individual treatment decisions. We therefore aimed to review and summarise all available safety data from controlled studies of the effect of ivermectin exposure in pregnancy to assist programmatic decision making and to better understand the implications of the use of ivermectin in pregnant women.

## Methods

### Search strategy and selection criteria

We did a systematic review and meta-analysis of ivermectin exposure during pregnancy. We searched MEDLINE, Scopus, Toxline, and the US FDA List of Pregnancy Exposure Registries on July 15, 2018. We did not use a language or date restriction. We also searched WHO’s International Clinical Trials Registry Platform, ClinicalTrials.gov, and the Cochrane Central Register of Controlled Trials, using the search terms “(ivermectin OR mectizan OR stromectol) AND pregnan*” and “(ivermectin OR mectizan OR stromectol) AND (abortion OR stillbirth OR malformation)”. The full search strategy is summarised in the appendix (p 3).

We included randomised controlled trials (RCTs) and observational studies, including cohort studies and case-control studies, that reported maternal or fetal serious and non-serious adverse events following oral administration of ivermectin to pregnant women at a dose of 150 μg/kg or more at any gestational timepoint. Background rates from pregnant women of the same or comparable population that had not received ivermectin (controls in cited studies) were used as a comparator (ie, the control group).

Expected serious adverse events in the context of this review included spontaneous abortions (death of the embryo or fetus before 28 weeks of gestation), stillbirths (the delivery of a baby that has died in the womb after at least 28 weeks of gestation), congenital anomalies, and neonatal death (the death of a baby before 28 days of age). Expected adverse events included maternal morbidity (weight loss, signs of ivermectin intoxication such as ataxia, tremor, and stupor), preterm births (delivery between 28 and 37 weeks resulting in a live baby), and low birthweight (term delivery of a baby weighing less than 2500 g).

Three review authors (WM, QB, and KCK) independently assessed the titles and abstracts of studies identified by the searches, and assessed the full-text copies for inclusion using a pre-piloted electronic eligibility form. If extracted data differed, the three review authors discussed these differences and, if unable to resolve them, involved other review authors (PN, CC, and AB) to reach consensus. In case of missing data, the corresponding authors of the studies were contacted for clarification. Multiple publications of the same trial were only included once. The extracted data included the study design, the study settings and population characteristics, context of the administration (eg, MDA programme for neglected tropical diseases), whether administration was inadvertent or intentional, ivermectin dosage and regimen, coadministration with another drug, and estimated gestational age at administration. Data for the number and description of both serious adverse events and adverse events were extracted for each study as well as number of events for the intervention and control groups, and total number of participants.

### Data analysis

The primary outcome measure was adverse pregnancy outcome (stillbirth, spontaneous abortions, or congenital malformations). A woman can have more than one outcome per pregnancy—ie, with multiple births or with stillbirth and malformation in a singleton pregnancy Meta-analysis of the serious adverse events was done separately for observational studies and RCTs, and was stratified by the type of serious adverse event. The risk of serious adverse events occurring in pregnant women exposed and non-exposed to ivermectin was estimated using odds ratios (ORs) as a pooled measure of effect. Reasons for substantial heterogeneity were explored using subgroup analysis of studies that had administered ivermectin in combination with albendazole—also a known teratogen in rats and rabbits and classified as FDA pregnancy category C^24^—which is common practice during lymphatic filariasis MDAs, and studies that had administered ivermectin alone. A random-effects model was chosen given the nature of the outcome being a rare event. Forest plots were used to present the pooled ORs and 95% CIs. Statistical heterogeneity was assessed using *I^2^*, which indicates the percentage of variation among the studies that occurs as a result of heterogeneity rather than chance. Variation across all studies was categorised as low (*I^2^* <25%), moderate (*I*^2^ between 50% and 75%), high (*I*^2^ >75%), or no statistical heterogeneity (*I^2^*= 0%).

Two review authors (PN and MFM) independently assessed the risk of bias for each included study using the Newcastle-Ottawa Scale for assessing the quality of observational studies.^25^ Risk of bias for RCTs was assessed using the Cochrane Risk of Bias Tool.^26^ The Newcastle-Ottawa Scale was used to evaluate the selection of participants, comparability of study groups, and the ascertainment of exposure or outcome of interest. The scale is grouped into three parts: selection (4 points), comparability (2 points), and outcome (3 points), for a maximum of 9 points. Studies scoring zero in any of the categories were classified as having high risk of bias. Studies scoring 1 point in any of the categories were classified as having moderate risk of bias and those scoring 2 points or more in all categories were classified as having low risk of bias.

Two separate sensitivity analyses were done on the primary outcome to test the robustness of the results by verifying that the overall effect estimates did not change after removing studies with high risk of bias and studies with fewer than 100 participants from the meta-analysis.

The certainty of the evidence was rated for each outcome using the GRADE approach.^27^ Evidence from RCTs starts at high quality, whereas evidence from observational data is considered low quality. The certainty in the evidence can be downgraded for risk of bias, imprecision, inconsistency, indirectness, and publication bias. Studies can also be upgraded if there was a large effect, a dose−response effect, and if all plausible residual confounding would reduce a demonstrated effect or would suggest a spurious effect if no effect was observed.^28^

The extracted data were entered and analysed using RevMan (version 5.3). The search and analysis protocol were registered on PROSPERO in 2016 (CRD42016046914).

### Role of the funding source

The funder of the study had no role in study design, data collection, data analysis, data interpretation, or writing of the report. The corresponding author had full access to all the data in the study and had final responsibility for the decision to submit for publication.

## Results

**Figure 1:**
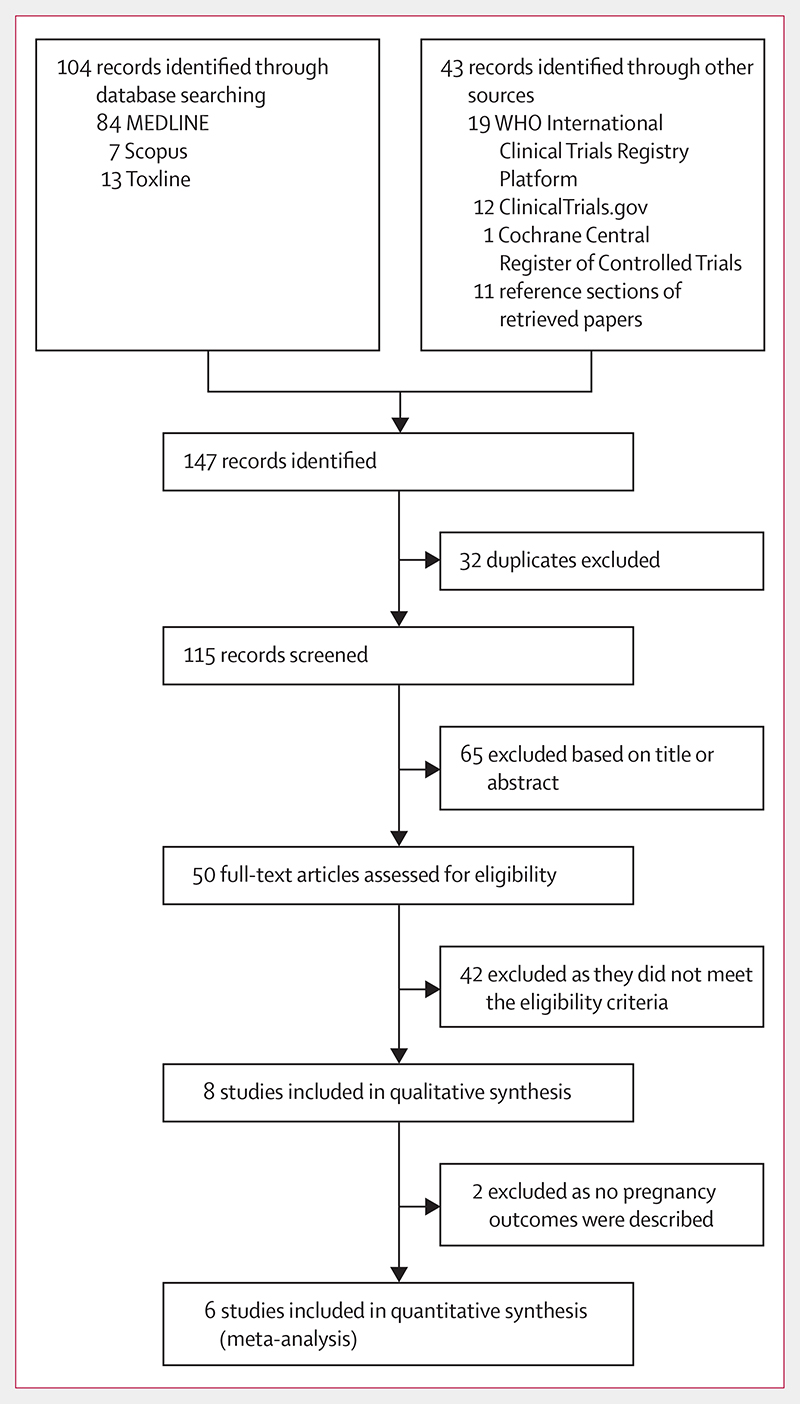
Flowchart showing the study selection process for the qualitative and quantitative synthesis

The initial search retrieved 147 records, of which only eight (5%) articles met the criteria for qualitative synthesis and six (4%) for quantitative analysis. Figure 1 depicts the study selection process according to the PRISMA statement for systematic reviews and meta-analysis.^29^ Despite contacting the corresponding authors of two eligible studies,^30,31^ we were unable to retrieve data for the number of events in the intervention and control groups needed for the meta-analysis and thus only analysed them qualitatively. Burnham^30^ described an RCT to determine adverse reactions to ivermectin given annually for treatment of onchocerciasis. Three pregnant women were inadvertently treated with ivermectin during the course of the trial, whose course of pregnancy and delivery was normal and no abnormality was noted in the children. Pregnancy outcomes in the control group were not described. Yumkella^31^ studied knowledge, attitudes, and practices regarding onchocerciasis with a focus on the perceptions of women during mass treatment campaigns with ivermectin. After drug distribution, 100 pregnant women were interviewed, and 27 reported having been inadvertently treated with ivermectin. No further information about pregnancy outcomes was provided.

The six studies included for the quantitative analysis were published between 1990 and 2008, and were done in six African countries: Uganda, Ghana, Cameroon, Tanzania, Mali, and Liberia (table 1). They encompassed a total of 893 women with 899 pregnancy outcomes; 496 pregnant women (500 pregnancy outcomes) received ivermectin inadvertently during MDA campaigns reported in nested case-control studies,^13,32–35^ and 397 pregnant women (399 pregnancy outcomes) purposely received ivermectin as part of an open-label RCT.^36^ The studies reported the following serious adverse events during pregnancy: spontaneous abortions, stillbirth, and congenital anomalies. Other serious adverse events and adverse events defined in the protocol were not described in these studies and therefore could not be included in the analysis.

97 women were reportedly exposed to ivermectin during the first trimester^32^ and 397 women during the second or third trimester.^36^ The time of exposure of the remaining 399 women was undefined after review and contact with the authors.^13,33–35^ The control group comprised pregnant women in the same population excluded from MDA or unexposed to ivermectin during the same period. Two of the retrospective case-control studies^34,35^ nested in the MDA campaigns and the RCT^36^ administered ivermectin and albendazole to pregnant women whereas all the other studies^13,32,33^ administered ivermectin alone. Excluded studies and rationale for exclusion after abstract or fulltext reading are presented in the appendix (pp 4, 5). Additionally, the sources of funding for included studies are detailed in the appendix (p 6).

None of the studies scored the maximum score of three points for selection bias (table 2). Pregnancy stage at time of ivermectin exposure was based on record linkage and retrospective self-reports. Contrary to the only RCT, none of the observational studies tested for pregnancy at time of ivermectin exposure; therefore, independent validation was insufficient. The case-control studies nested in the MDA campaigns were not designed to answer whether ivermectin is safe during pregnancy, hence comparability between ivermectin-exposed and control groups was poorly matched for important factors likely to bias the primary outcome, such as age of the mother, risk behaviour, history of pregnancy-related adverse events, distance from the participant’s home to a health-care facility, or any other important factor. Only Pacque and colleagues^13^ ensured similar age groups were included in both groups at the time of analysis, while Makene and colleagues^35^ ensured abnormalities such as splenomegaly and associated changes commonly expected in areas of high endemicity for malaria and other infections were common to both groups. Two studies^33,34^ relied on self-reports of serious adverse events rather than health facility records. The ascertainment of exposure of cases and controls was equally poor in all studies. Only one study^13^ reported participant record linkage during MDA through a house-to-house census, whereas other studies relied on self-reports of drug intake. Overall the risk of bias was high because three of the five studies did not score on comparability.

The risk of bias for Ndyomugyenyi and colleagues’ study^36^ was assessed separately using the Cochrane risk-of-bias tool for RCTs.^26^ The risk of bias was judged as high because of an undescribed allocation concealment method and the absence of blinding, which might have increased performance bias (table 3).

**Figure 2:**
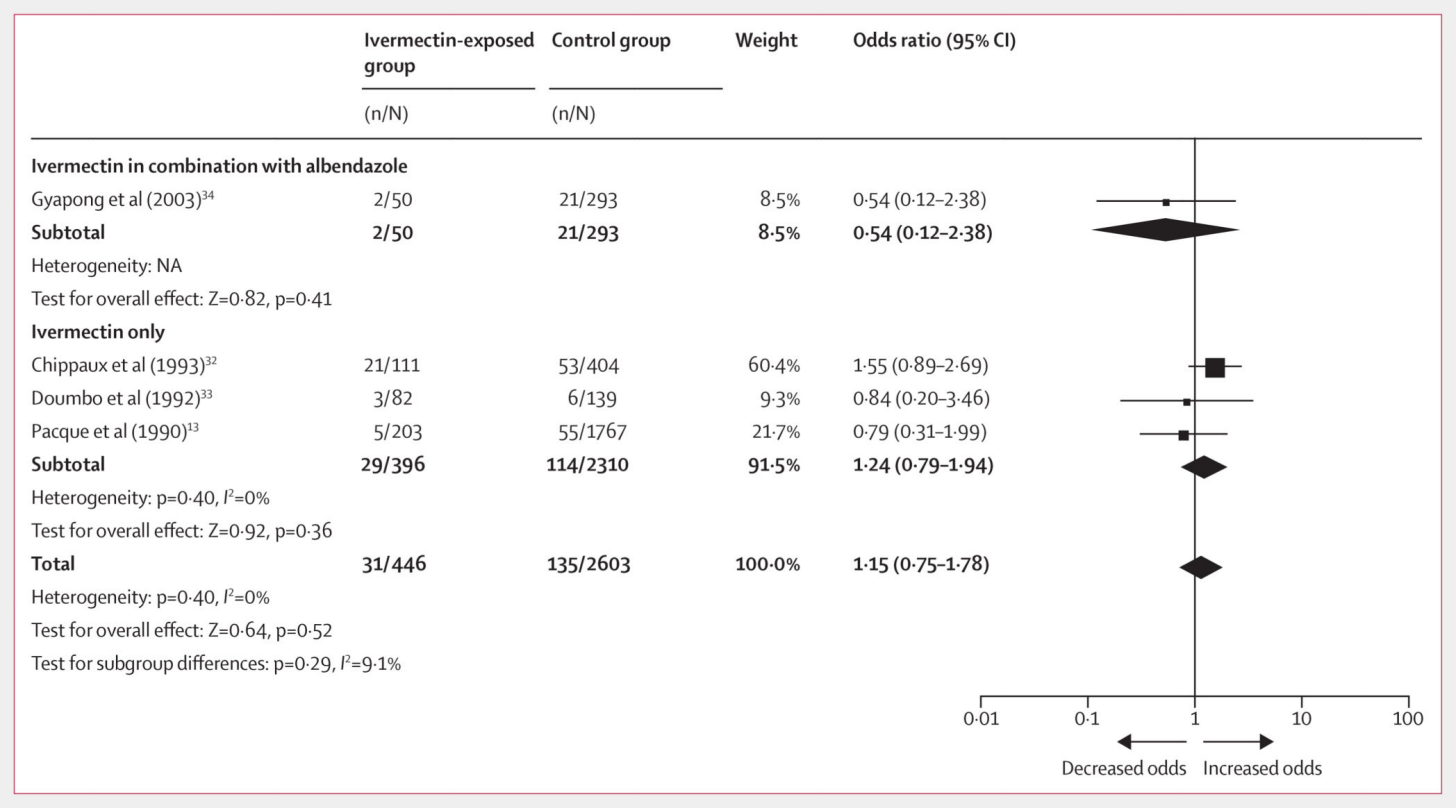
Forest plot for risk of spontaneous abortions and stillbirths after exposure to ivermectin during pregnancy compared with no exposure Evidence is from observational studies. NA=not applicable.

The observational studies reported 31 spontaneous abortions and stillbirths from 446 outcomes of pregnancies inadvertently exposed to ivermectin compared with 135 cases from 2603 control outcomes (OR 1·15, 95% CI 0·75−1·78; figure 2; table 4). Subgroup analysis on the concomitant administration of albendazole and ivermectin showed no significant odds of spontaneous abortions and stillbirths (0·54, 0·12−2·38; p=0·41) as with ivermectin alone (1·24, 0·79−1·94; p=0·36; figure 2). The RCT^36^ reported four spontaneous abortions and stillbirths from 399 pregnancy outcomes after exposure to ivermectin or ivermectin in combination with albendazole during the second and third trimester, compared with seven events from 438 pregnancy outcomes in the control group (0·62, 0·18−2·14; table 5).

**Figure 3:**
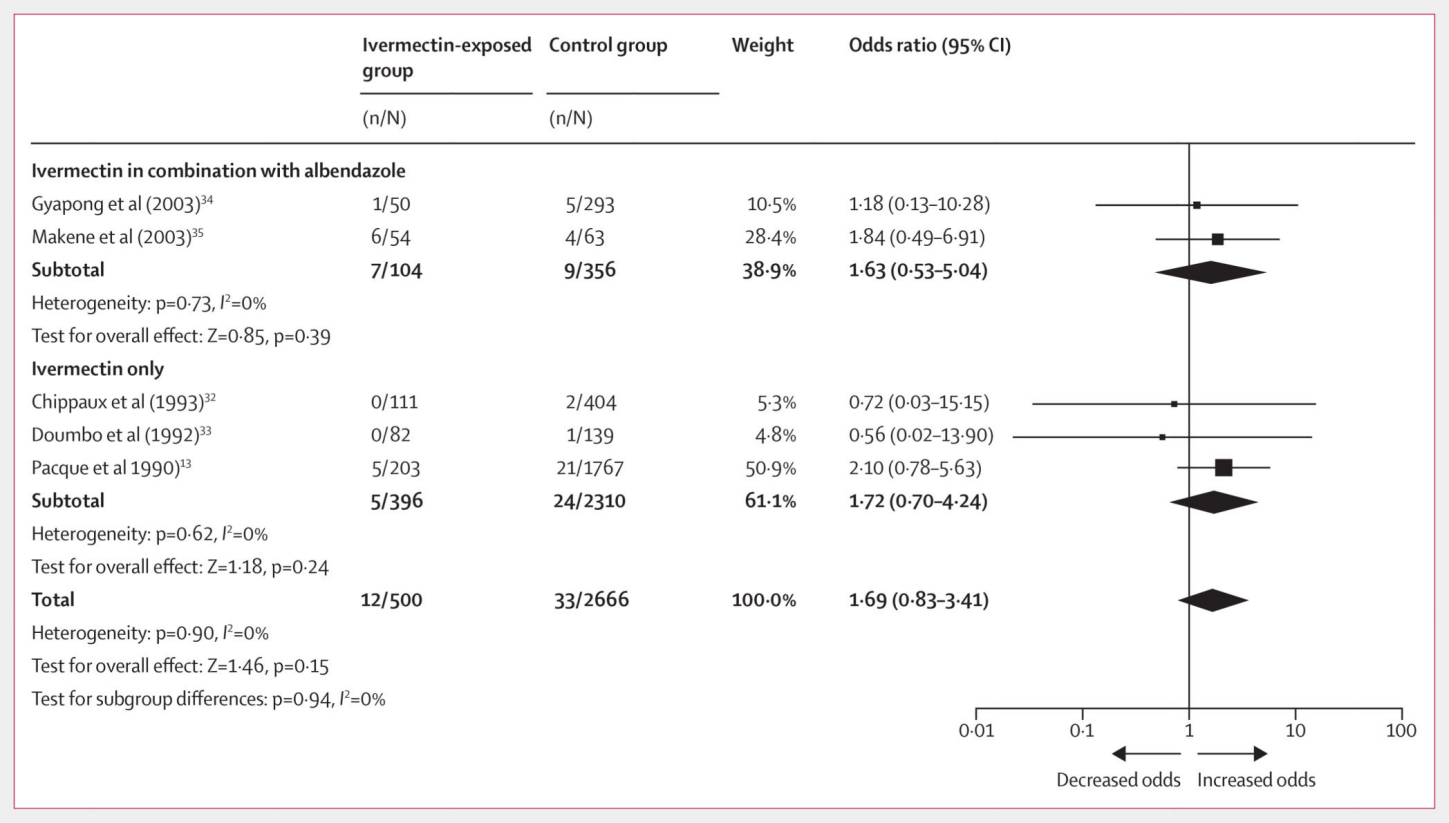
Forest plot for risk of congenital anomalies after exposure to ivermectin during pregnancy compared with no exposure Evidence is from observational studies.

Additionally, the observational studies reported 12 congenital anomalies from 500 pregnancy outcomes compared with 33 cases from 2666 control outcomes (OR 1·69, 95% CI 0·83−3·41; figure 3; table 4). The certainty of this evidence from observational studies was assessed as very low using the GRADE approach; the details of each component of the assessment are provided in the appendix (p 7).

Subgroup analysis on the safety of ivermectin in combination with albendazole revealed similar odds of congenital anomalies after receiving ivermectin in combination with albendazole (OR 1·63, 95% CI 0·53−5·04) compared with ivermectin alone (1·72, 0·70−4·24). The RCT^36^ reported only one congenital anomaly from 399 pregnancy outcomes of women inadvertently exposed to ivermectin or ivermectin in combination with albendazole during their second and third gestational trimesters as well as one case from 438 outcomes from the albendazole group (1·10, 0·07−17 · 65; table 5). The certainty of the evidence from the RCT was assessed as very low using the GRADE approach; the details of each component of the assessment are provided in the appendix (p 8).

Publication bias was not assessed because there were less than ten studies included in this systematic review and meta-analysis.

Sensitivity analyses were done when possible. After excluding studies at high risk of bias^32–34^ from the metaanalysis describing the risk of congenital anomalies, the overall point estimate did not change significantly (from OR 1·69 [95% CI 0·83−3·41] to 2·0 [0·91−4·42]). The same analysis was not possible for spontaneous abortions and stillbirths because three out of five studies were considered to be at high risk of bias. After excluding trials with wide confidence intervals and low number of events^33,34^ from the meta-analysis, the odds of spontaneous abortions and stillbirths did not change significantly (from OR 1·15 [95% CI 0·75−1·78] to 1·22 [0·65−2·30]), nor did the odds of congenital anomalies (from 1·69 [0·83−3·41] to 12·9 [0·74−4·85]).

## Discussion

Although serious adverse events were reported during pregnancy in a non-negligible frequency (1·36% in observational studies and 0·6% in one RCT), any causal relationship between ivermectin administration and the unfavourable clinical outcome cannot be easily assessed, since the number of recorded exposures is too low to achieve statistical power and it is not possible to discard selection bias due to absence of blinding and randomised controls.

Only eight papers were eligible for inclusion in our review. These studies encompass 893 women exposed inadvertently to ivermectin during pregnancy; of these, only 97 were reportedly exposed during the first trimester. Pooled results from all nested retrospective case-control studies showed no difference in pregnancy-related serious adverse events from inadvertently exposed mothers. The only RCT included showed a non-significant effect of ivermectin exposure in pregnancy on increased rates of abortions, stillbirths, and congenital anomalies. Overall, given the small sample, point estimates of serious adverse events had wide overlapping CIs crossing the point of no effect.

The results of the primary outcomes were graded as very low certainty of evidence because of bias generated by improper study designs and lack of power leading to a high degree of imprecision. The review authors downgraded observational studies mainly because of comparability bias, as studies were unable to ensure that the pregnant women between study groups were comparable in regard to key risk factors such as age and history of serious adverse events during pregnancy. The lack of comparability is unsurprising given that these studies were not designed as case-control studies to address safety of ivermectin during pregnancy but were reports of observations following MDA programmes. Evidence from the RCT was also rated as very low certainty of evidence, as the study was not blinded (risk of performance bias) and we could not clearly assess the allocation concealment method (risk of selection bias). Additionally, all studies were underpowered, estimates had wide confidence intervals, very few events were recorded, and the point estimate included the point of no effect. Importantly, given that ivermectin exposure was determined based on record linkage and retrospective self-reports, the potential role of recall bias cannot be determined.

The included studies were not adequately designed to address the question posed in this review of whether ivermectin exposure could negatively affect pregnancy outcomes. During the first trimester, women are less likely to reveal their state because of social risk, desire for privacy, and doubts, and hence are potentially more exposed to inadvertent treatment. However, fewer than 100 known exposures to ivermectin in the first trimester were identified and included in this meta-analysis.

Given the absence of evidence to support clinical trials with ivermectin in pregnancy, plausible next steps could include reproductive toxicological studies in primates. Another readily available option is an open data repository of inadvertent drug exposures during pregnancy. We estimate that given a baseline population rate of congenital anomalies of 23·9 per 1000 births,^37^ a sample of at least 72 000 exposures is needed to detect a 10% increase due to ivermectin (80% power at 5% significance). For stillbirths, this number increases to 92 000 given a baseline rate of 18·3 per 1000 births.^38^ These numbers, although very large, do still appear feasible if one considers that more than 300 million people are treated every year in the context of onchocerciasis and lymphatic filariasis elimination programmes. However, despite large-scale MDA with ivermectin over the past 30 years, there are very few records of well documented outcomes after inadvertent exposure to ivermectin in pregnancy. These available records are from African populations, extracted from underpowered studies with a design not intended for this purpose. Given the frequency and distribution of MDA programmes of ivermectin, it is remarkable that no reports have been published in the past 10 years. This study cannot draw evidence-based conclusions on whether or not there are deleterious effects of oral ivermectin during pregnancy. Further high-quality evidence supporting the safety of ivermectin administration in this particular vulnerable group is imperative.

This review was limited by the small number of published reports available and the fact that all included studies were done more than 10 years ago with some going back almost 30 years; only a few of the corresponding authors contacted were able to respond to requests for additional details.

When ivermectin is used in MDA for onchocerciasis control, population coverage is a key factor for effectiveness;^39^ a similar community effect is expected for the proposed new indication to reduce malaria transmission.^40,41^ Reproductive toxicological studies of primates might provide further insight on the safety of ivermectin during pregnancy in addition to the development of an open and high-quality data repository on the outcome of inadvertently exposed pregnancies.

## Supplementary Material

Supplementary appendix

## Figures and Tables

**Table 1:** Summary of included studies for the quantitative analysis

	Study design	Country	Inadvertent treatment	Concomitant albendazole	Gestational period	Spontaneous abortions and stillbirths		Congenital anomalies	
						Ivermectin-exposed group	Control group	Ivermectin-exposed group	Control group
Chippaux et al (1993)^32^	Retrospective case-control study	Cameroon	Yes, during MDA programme	No	First trimester*	21/111	53/404	0/111	2/404
Doumbo et al (1992)^33^	Retrospective case-control study	Mali	Yes, during MDA programme	No	Unclear	3/82	6/139	0/82	1/139
Gyapong et al (2003)^34^	Retrospective case-control study	Ghana	Yes, during MDA programme	Yes	Unclear	2/50	21/293	1/50	5/293
Makene et al (2003)^35^	Retrospective case-control study	Tanzania	Yes, during MDA programme	Yes	Unclear	NA	NA	6/54	4/63
Ndyomugyenyi et al (2008)^36^	Open-label randomised controlled trial	Uganda	No	Yes	Second and third trimester	4/399	7/438	1/399	1/438
Pacque et al (1990)^13^	Retrospective case-control study	Liberia	Yes, during MDA programme	No	Unclear	5/203	55/1767	5/203	21/1767

Data are n/N, unless otherwise specified. MDA=mass drug administration. NA=not available. *97 of 110 were exposed during the first trimester of pregnancy; the remaining 13 were not specified. These 110 exposures generated 111 pregnancy outcomes.

**Table 2:** Risk of bias assessment of the observational studies using the Newcastle-Ottawa Scale

	Selection*		Comparability†		Exposure‡		Risk of bias
	Score	Notes	Score	Notes	Score	Notes	
Chippaux et al (1993)^32^	2	Pregnancy tests were not done and therefore no independent validation of pregnancy was available	NA	Study was not controlled for age, history of pregnancy-related serious adverse events, or any other substantial factor	2	Exposure to ivermectin was self-reported	High
Doumbo et al (1992)^33^	2	Pregnancy tests were not done and therefore no independent validation of pregnancy was available; adverse pregnancy outcomes were self-reported	NA	Study was not controlled for age, history of pregnancy-related serious adverse events, or any other substantial factor	2	Exposure to ivermectin was self-reported	High
Gyapong et al (2003)^34^	2	Pregnancy tests were not done and therefore no independent validation of pregnancy was available	NA	Study was not controlled for age, history of pregnancy-related serious adverse events, or any other substantial factor	1	Exposure to ivermectin was self-reported; the study reports exposure to albendazole or ivermectin during pregnancy, in which it is unclear if all cases received ivermectin	High
Makene et al (2003)^35^	2	Pregnancy tests were not done and therefore no independent validation of pregnancy was available; adverse pregnancy outcomes were self-reported	1	..	2	Ascertainment of exposure was not described	Moderate
Pacque et al (1990)^13^	2	Pregnancy tests were not done and therefore no independent validation of pregnancy was available	1	..	3	..	Moderate

NA=not available. *Maximum score of 4. †Maximum score of 2. ‡Maximum score of 3.

**Table 3:** Risk of bias assessment of Ndyomugyenyi et al (2008)^36^ using the Cochrane risk-of-bias tool for randomised controlled trials

	Risk of bias	Support for judgment
Random sequence generation and allocation concealment (selection bias)	Unclear	A random sequence was generated in SPSS; the allocation concealment method was not described
Blinding of participants and personnel (performance bias)	High	The study design was an open-label randomised controlled trial
Blinding of outcome assessment (detection bias)	Low	Severe adverse events are an objective outcome and their detection is unlikely to have been affected by no blinding
Incomplete outcome data (attrition bias)	Low	Loss to follow-up was similar across the different study groups, ranging from 26% to 33%
Selective outcome reporting (reporting bias)	Unclear	A study protocol was not found in any of the clinical trial registries; the study was not registered in any clinical trial repository
Other bias	Low	The authors took measures to prevent baseline imbalances between study groups

**Table 4:** Summary of data from the observational studies measuring serious adverse events in women exposed to ivermectin during pregnancy

	Studies	Participants	Observations for inadvertent exposure to ivermectin during pregnancy	Observations among pregnant women who did not receive ivermectin	Serious adverse events among women who received ivermectin during pregnancy	Serious adverse events among pregnant women in the control group	Weighted odds ratio (95% CI)
Spontaneous abortions and stillbirths	4	3042	446	2603	31	135	1·15 (0·75−1·78)
Congenital anomalies	5	3159	500	2666	12	33	1·69 (0·83−3·41)

Data are n unless stated otherwise. All studies were retrospective case-control studies. The number of pregnancy outcomes exceeds the number of pregnant women because of several sets of twins.

**Table 5:** Summary of data from the single randomised controlled trial measuring serious adverse events in women treated with ivermectin during pregnancy

	Studies	Participants	Observations for inadvertent exposure to ivermectin during pregnancy	Observations among pregnant women who did not receive ivermectin	Serious adverse events among women who received ivermectin during pregnancy	Serious adverse events among pregnant women in the control group	Odds ratio (95% CI)
Spontaneous abortions and stillbirths	1	832	399	438	4	7	0·62 (0·18−2·14)
Congenital anomalies	1	832	399	438	1	1	1·10 (0·07−17·65)

Data are n unless stated otherwise. The number of pregnancy outcomes exceeds the number of pregnant women because of several sets of twins.

## Research in context

### Evidence before this study

Ivermectin is one of the most broadly used drugs in global health. More than 3·7 billion treatments have been safely distributed in the context of Merck’s donation programme. Pregnant women are usually excluded from treatment but no formal evaluation of the safety in pregnancy has been done to date. Additionally, no systematic reviews or meta-analyses of ivermectin exposure in pregnant women have been published according to our provisional literature search of MEDLINE, Scopus, and the Cochrane Library.

### Added value of this study

Pregnant women are getting inadvertently exposed to ivermectin during mass drug administration campaigns. Weighing the risks and benefits of ivermectin in pregnancy is crucial for informed public health policies and for individual treatment decisions. In this systematic review and meta-analysis, we determined the odds ratio of adverse pregnancy outcomes after exposure to ivermectin during pregnancy compared with untreated pregnant women from matching populations in controlled studies. We identified an important evidence gap regarding the effect of ivermectin exposure in pregnancy.

### Implications of all the available evidence

High-quality evidence supporting the safety of ivermectin administration in this vulnerable group is imperative. The existing data have been generated in studies not designed to determine the safety of ivermectin administration during pregnancy. Further steps for generating the necessary safety data should encompass an open data repository of inadvertent drug exposures during pregnancy among other potential readily available options.
