# Supplementary material for: Safety of oral ivermectin during pregnancy: a systematic review and meta-analysis
Source: Lancet Glob Health. Author manuscript; Available in PMC 2022 Sep 5. (PMC7613514; doi:10.1016/S2214-109X(19)30453-X)
Supplement: Supplementary appendix [file EMS153500-supplement-Supplementary_appendix.pdf]

THE LANCET
Global Health

Supplementary appendix

This appendix formed part of the original submission and has been peer reviewed. We post it as supplied by the authors.

Supplement to: Nicolas P, Maia MF, Bassat Q, et al. Safety of oral ivermectin during pregnancy: a systematic review and meta-analysis. *Lancet Glob Health* 2020;
8: e92–100.

Protocol deviations

In the protocol, the main outcomes described as maternal morbidity attributable to the drug include: abortions, pre-term births, stillbirths, low birth weight, congenital anomalies, neonatal death, infant survival up 2 years of age, hospital admissions in the first two years of life, and developmental milestones in the first two years of life. Because of the limited evidence available for such a broad number of variables with different severity, pregnancy outcomes have been sub grouped as severe adverse events and adverse events following FDA definition for a more accurate analysis based on severity.

Therefore, in this review abortion, stillbirth, congenital malformations, and neonatal death are considered SAE; non-severe maternal morbidity, pre-term births, and low birth weight are classified as AE.

Infant survival, hospital admissions and developmental milestones in the first two years of life have been dismissed in order to address the analysis to pregnancy outcomes that can be measured during pregnancy or in a short time after delivery.

Risk of bias assessment for observational studies is not described in the protocol; during the development of the review the Newcastle-Ottawa scale (NOS) was used to assess the risk of bias of observational studies found. However, for the one RCT found, the Cochrane tool for risk of bias assessment was used as described in the protocol.

Finally, the McMaster quality assessment scale of harms tool was included in registered protocol to evaluate the quality of adverse pregnancy outcomes, however this tool was not used due to the low number of severe adverse events found in the review.

Supplementary Table 1: Reproductive toxicity studies submitted by Merck as part of the New Drug Application of ivermectin

**Species**	**Timing**	**Daily dose**	**Cumulative dose**	**Adverse maternal effect**	**Adverse pregnancy outcome**
Mice (20-25/group)	Gestation days 6-15[a]	0, 0.1 mg/kg/day	0-1 mg/kg in 10 days	Not found	Not found
		0.2 mg/kg/day	2mg/kg in 10 days	Tremors and convulsions	Not found
		0.4 mg/kg/day	4mg/kg in 10 days	+ Some maternal deaths	Teratogenicity Cleft palate
		0.8 mg/kg/day	8 mg/kg in 10 days	As above	+ exencephaly
		1.6 mg/kg/day	16 mg/kg in 10 days		
Rats (25/group)	Gestation days 6-17[b]	0 mg/kg/day	-	Not found	Not found
		2.5 mg/kg/day*	30 mg/kg in 12 days		
		5 mg/kg/day	60 mg/kg in 12 days	Not found	Incomplete bone ossification
		10 mg/kg/day	120 mg/kg in 12 days	Some maternal deaths	+ cleft palate and wavy ribs
Rabbits (16/group)	Gestation days 6-18[c]	0 mg/kg/day	-	Not found	Not found
		1.5 mg/kg/day	19.5 mg/kg in 13 days		
		3 mg/kg/day	39 mg/kg in 13 days	Not found	Cleft palate Clubbed forepaws
		6 mg/kg/day	78 mg/kg in 13 days	Body weight loss	+ abortions and stillbirths
**Primates**	Not done/no available data				

Supplementary Table 2: Search Strategy

MEDLINE (PubMed)

#1	(ivermectin OR mectizan OR stromectol)
#2	#1 AND (pregnan* OR gestation)
#3	#2; Filters: Humans
#4	#3; Filters: Clinical Trial
#5	#3 AND abortion
#6	#5 OR stillbirth
#7	#3 AND (malformation OR congenital anomaly)
#8	#3 AND (low birth weight OR prematurity OR death)
#9	#3 AND adverse events

Toxnet

#1	(ivermectin OR mectizan OR stromectol)
#2	#1 AND pregnan*
#3	#2 AND human
#4	#2 AND stillbirth
#5	#2 AND malformation
#6	#2 AND congenital malformation

Scopus

#1	(ivermectin OR mectizan OR stromectol)
#2	#1 AND pregnan*
#3	#2 Filters: Human, Clinical Trials
#4	#3 AND stillbirth
#5	#3 AND malformation
#6	#3 AND congenital malformation

Supplementary Table 3: Characteristics of excluded studies (after abstract/full-text reading)

Reference	Comment
Abengunde *et al.* 2016 [1]	Does not include pregnant women.
Alout *et al.* 2014 [2]	Study is based on field entomological assessments.
Ashraf *et al.* 2016 [3]	In vitro study.
Bialek *et al.* 1999 [4]	Provides general information about the negative effect of ivermectin in pregnancy based on preclinical evidence.
Biritwum *et al.* 1997 [5]	Does not include pregnant women.
Blackburn *et al.* 2006 [6]	Does not include pregnant women.
Brieger *et al.* 2002 [7]	Does not include pregnant women.
Cartel *et al.* 1992 [8]	Does not include pregnant women.
Chippaux *et al.* 1995 [9]	Evaluates different methods of pregnancy screening during ivermectin MDA.
Cook *et al.* 1995 [10]	Review that provides general information about adverse effects of chemotherapeutic agents used in tropical medicine.
Cupp *et al.* 2004 [11]	Assesses the effects of long-term community level treatment with ivermectin (Mectizan) on adult *Onchocerca volvulus*.
Da Silva *et al.* 1997 [12]	Review that provides information of clinical pharmacology of anthelmintic drugs.
Dupouy-Camet *et al.* 2003 [13]	Provides information about ivermectin use in tropical medicine and the general safety for pregnant women is discussed.
Einsiedel *et al.* 2008 [14]	Does not include pregnant women.
Fawcett *et al.* 2003 [15]	Provides information about ivermectin use in scabies and concludes that ivermectin safety in pregnant women has to be established.
Guderian *et al.* 1997 [16]	Describes spontaneous abortions in areas of ivermectin MDA without direct treatment of pregnant women.
Hengge *et al.* 2006 [17]	Provides general information of scabies.
Henriquez-Camacho *et al.* 2016 [18]	Does not include pregnant women.
Executive Committee of Guideline for the Diagnosis of scabies in Japan *et al.* 2008 [19]	Guidelines that indicate that ivermectin safety is not established for pregnant women.
Johnston *et al.* 2005 [20]	Does not include pregnant women.
Kearns *et al.* 2015 [21]	Does not include pregnant women.
Kearns *et al.* 2017 [22]	Does not include pregnant women.
Krolewiecki *et al.* 2013 [23]	Review that indicates that ivermectin is contraindicated in pregnant women.
Lawrence *et al.* 2005 [24]	Does not include pregnant women.

Masud *et al.* 2009 [25]	Does not include pregnant women.
Osei-Atweneboana *et al.* 2007 [26]	Does not include pregnant women.
Osei-Atweneboana *et al.* 2011 [27]	Commentary/review.
Ottesen *et al.* 2008 [28]	Evaluates the impact of the global programme to eliminate lymphatic filariasis. Mentions the contraindication of ivermectin during pregnancy.
Pacque *et al.* 1989 [29]	Does not include pregnant women.
Potasman *et al.* 1998 [30]	Case report about a pregnant traveller that ingested ivermectin and other drugs and had an elective abortion.
Richard-Lenoble *et al.* 2003 [31]	Provides general information about anti-parasitic treatments in pregnant women.
Richard-Lenoble *et al.* 2003 [32]	Provides general information about ivermectin and filariasis, and mentioned that ivermectin is contraindicated in pregnant women.
Rosenblatt *et al.* 1992 [33]	Provides anti-parasitic agents information.
Rosenblatt *et al.* 1999 [34]	Provides anti-parasitic agents information.
Sheele *et al.* 2013 [35]	Does not include pregnant women.
Soungalo *et al.* 1997 [36]	Does not include pregnant women
Stephenson *et al.* 2000 [37]	Provides general information on anthelmintic treatment.
Taylor et al. 2010 [38]	General review on lymphatic filariasis and onchocerciasis.
Taylor *et al.* 2014 [39]	Does not include pregnant women.
Vaidhyanathan *et al.* 2001 [40]	Review of ivermectin in scabies, mentions that safety in pregnant women must be established.
Walker *et al.* 2000 [41]	Review of Interventions for treating scabies.
Whitworth *et al.* 1996 [42]	Does not include pregnant women.

Supplementary Table 4: Source of funding

**Reference**	**Source of funding**
Chippaux *et al.* 1993	This work received financial support from the UNDPiWorld Bank/WHO Special Programme for Research and Training in Tropical Diseases (ID no. 870336)
Doumbo *et al.* 1992	Onchocerciasis Controle Programme (OCP), Département d'Épidémiologie des Affections Parasitaires (DEAP) de l'École Nationale de Médicine et Pharmacie du Mali (ENMP), Project MOS/OCP/OCT nº 86010)
Gyapong *et al.* 2003	This investigation received financial support from the UNDP/World Bank/WHO Special Programme for Research and Training in Tropical Diseases and GlaxoSmithKline
Makene *et al.* 2003	Not available
Ndyomugyenyi *et al.* 2008	Funding for the study was provided by DBL- Centre for Health Research and Development. The American Society of Tropical Medicine and Hygiene (ASTMH) assisted with publication expenses
Pacque *et al.* 1990	This study was supported by funds by the UNDP/World Bank/WHO Special Programme for Research and Training in Tropical Diseases (ID no 870096 and TDR 890525) and the National Institutes of Health (ID no S10-RR04060)

Supplementary Table 5: certainty of assessment using GRADE for observational studies

Certainty of assessment using GRADE approach[1]	Risk of bias	Inconsistency	Indirectness	Imprecision	Certainty of evidence (GRADE)	Comments
Spontaneous abortions and stillbirths	HIGH Downgrade by 1[2]	LOW No downgrade[3]	LOW No downgrade[4]	HIGH Downgrade by 1[5]	VERY LOW ⊕⊖⊖⊖	There is not enough evidence to assess whether ivermectin increases the risk of abortions and stillbirths.
Congenital anomalies	HIGH Downgrade by 1[2]	LOW No downgrade[3]	LOW No downgrade[4]	HIGH Downgrade by 1[5]	VERY LOW ⊕⊖⊖⊖	We do not know if the exposure to ivermectin during pregnancy increases the risk of congenital anomalies.

Legend:

[1] Observational studies start as "low quality of evidence".

[2] Considerable risk of bias was detected using the Newcastle-Ottawa scale (NOS) (See Table 3).

[3] There was no statistical heterogeneity (I^2 = 0%).

[4] The studies were all conducted in a way that directly addresses the review question.

[5] We downgraded for imprecision given the low number of events in the group that had received ivermectin during pregnancy and the pooled estimate crossed the point of no effect (OR=1) and significant harm.

Supplementary Table 6: certainty of assessment using GRADE for RCT

Certainty of assessment using GRADE approach[1]	Risk of bias	Inconsistency	Indirectness	Imprecision	Certainty of evidence (GRADE)	Comments
Spontaneous abortions and stillbirths	HIGH Downgrade by 1[2]	LOW No downgrade[3]	LOW No downgrade[4]	HIGH Downgrade by 1[5]	VERY LOW ⊕⊖⊖⊖	We do not know if the exposure to ivermectin during pregnancy increases the risk of abortions and stillbirths.
Congenital anomalies	HIGH Downgrade by 1[2]	LOW No downgrade[3]	LOW No downgrade[4]	HIGH Downgrade by 1[5]	VERY LOW ⊕⊖⊖⊖	We do not know if the exposure to ivermectin during pregnancy increases the risk of congenital anomalies.

Legend:

[1] Randomised controlled trials start off as high quality of evidence.

[2] Risk of bias was judged as high because of an undescribed allocation concealment method and the absence of blinding, which may have increased performance bias.

[3] Only one RCT was included.

[4] The study directly addresses the review question.

[5] Downgraded for imprecision because the study was underpowered to measure a difference in adverse events between study arms. The numbers of participants and events were very low, the point estimates for both risk of spontaneous abortion and stillbirths and congenital anomalies crossed both the point of no effect (OR=1) and substantial harm.

References

1. Abegunde AT, Ahuja RM, Okafor NJ. Doxycycline plus ivermectin versus ivermectin alone for treatment of patients with onchocerciasis. Cochrane Database Syst Rev. 2016;(1):CD011146. doi: 10.1002/14651858.CD011146.pub2. PubMed PMID: 26771164; PubMed Central PMCID: PMCPMC5029467.

2. Alout H, Krajacich BJ, Meyers JI, Grubaugh ND, Brackney DE, Kobylinski KC, et al. Evaluation of ivermectin mass drug administration for malaria transmission control across different West African environments. Malar J. 2014;13:417. doi: 10.1186/1475-2875-13-417. PubMed PMID: 25363349; PubMed Central PMCID: PMC4226880.

3. Ashraf S, Prichard R. Ivermectin exhibits potent anti-mitotic activity. Vet Parasitol. 2016;226:1-4. doi: 10.1016/j.vetpar.2016.06.015. PubMed PMID: 27514873.

4. Bialek R, Knobloch J. [Parasitic infections in pregnancy and congenital parasitoses. II. Helminth infections]. Z Geburtshilfe Neonatol. 1999;203(3):128-33. PubMed PMID: 10448707.

5. Biritwum RB, Sylla M, Diarra T, Amankwa J, Brika GP, Assogba LA, et al. Evaluation of invermectin distribution in Benin, Cote d'Ivoire, Ghana and Togo: estimation of coverage of treatment and operational aspects of the distribution system. Ann Trop Med Parasitol. 1997;91(3):297-305. PubMed PMID: 9229022.

6. Blackburn BG, Eigege A, Gotau H, Gerlong G, Miri E, Hawley WA, et al. Successful integration of insecticide-treated bed net distribution with mass drug administration in Central Nigeria. Am J Trop Med Hyg. 2006;75(4):650-5. PubMed PMID: 17038688.

7. Brieger WR, Otusanya SA, Oke GA, Oshiname FO, Adeniyi JD. Factors associated with coverage in community-directed treatment with ivermectin for onchocerciasis control in Oyo State, Nigeria. Trop Med Int Health. 2002;7(1):11-8. PubMed PMID: 11851950.

8. Cartel JL, Nguyen NL, Moulia-Pelat JP, Plichart R, Martin PM, Spiegel A. Mass chemoprophylaxis of lymphatic filariasis with a single dose of ivermectin in a Polynesian community

with a high Wuchereria bancrofti infection rate. Trans R Soc Trop Med Hyg. 1992;86(5):537-40. PubMed PMID: 1475825.

9. Chippaux JP, Gardon-Wendel N, Ernould JC, Gardon J. [Comparison between various methods of pregnancy screening during a large-scale ivermectin treatment in Cameroon]. Bull Soc Pathol Exot. 1995;88(3):129-33. PubMed PMID: 8555769.

10. Cook GC. Adverse effects of chemotherapeutic agents used in tropical medicine. Drug Saf. 1995;13(1):31-45. PubMed PMID: 8527018.

11. Cupp EW, Duke BO, Mackenzie CD, Guzman JR, Vieira JC, Mendez-Galvan J, et al. The effects of long-term community level treatment with ivermectin (Mectizan) on adult Onchocerca volvulus in Latin America. Am J Trop Med Hyg. 2004;71(5):602-7. PubMed PMID: 15569792.

12. de Silva N, Guyatt H, Bundy D. Anthelmintics. A comparative review of their clinical pharmacology. Drugs. 1997;53(5):769-88. PubMed PMID: 9129865.

13. Dupouy-Camet J, Yera H, Tourte-Schaefer C. [Ivermectin use in tropical medicine]. Arch Pediatr. 2003;10 Suppl 5:545s-9s. PubMed PMID: 15022779.

14. Einsiedel L, Fernandes L. Strongyloides stercoralis: a cause of morbidity and mortality for indigenous people in Central Australia. Intern Med J. 2008;38(9):697-703. doi: 10.1111/j.1445-5994.2008.01775.x. PubMed PMID: 19143887.

15. Fawcett RS. Ivermectin use in scabies. Am Fam Physician. 2003;68(6):1089-92. PubMed PMID: 14524395.

16. Guderian RH, Lovato R, Anselmi M, Mancero T, Cooper PJ. Onchocerciasis and reproductive health in Ecuador. Trans R Soc Trop Med Hyg. 1997;91(3):315-7. PubMed PMID: 9231206.

17. Hengge UR, Currie BJ, Jager G, Lupi O, Schwartz RA. Scabies: a ubiquitous neglected skin disease. Lancet Infect Dis. 2006;6(12):769-79. doi: 10.1016/S1473-3099(06)70654-5. PubMed PMID: 17123897.

18. Henriquez-Camacho C, Gotuzzo E, Echevarria J, White AC, Jr., Terashima A, Samalvides F, et al. Ivermectin versus albendazole or thiabendazole for Strongyloides stercoralis infection. Cochrane

Database Syst Rev. 2016;(1):CD007745. doi: 10.1002/14651858.CD007745.pub3. PubMed PMID: 26778150; PubMed Central PMCID: PMCPMC4916931.

19. Executive Committee of Guideline for the D, Ishii N. Guideline for the diagnosis and treatment of scabies in Japan (second edition). J Dermatol. 2008;35(6):378-93. doi: 10.1111/j.1346-8138.2008.00491.x. PubMed PMID: 18578720.

20. Johnston FH, Morris PS, Speare R, McCarthy J, Currie B, Ewald D, et al. Strongyloidiasis: a review of the evidence for Australian practitioners. Aust J Rural Health. 2005;13(4):247-54. doi: 10.1111/j.1440-1584.2005.00710.x. PubMed PMID: 16048468.

21. Kearns TM, Speare R, Cheng AC, McCarthy J, Carapetis JR, Holt DC, et al. Impact of an Ivermectin Mass Drug Administration on Scabies Prevalence in a Remote Australian Aboriginal Community. PLoS Negl Trop Dis. 2015;9(10):e0004151. doi: 10.1371/journal.pntd.0004151. PubMed PMID: 26516764; PubMed Central PMCID: PMCPMC4627839.

22. Kearns TM, Currie BJ, Cheng AC, McCarthy J, Carapetis JR, Holt DC, et al. Strongyloides seroprevalence before and after an ivermectin mass drug administration in a remote Australian Aboriginal community. PLoS Negl Trop Dis. 2017;11(5):e0005607. doi: 10.1371/journal.pntd.0005607. PubMed PMID: 28505198; PubMed Central PMCID: PMCPMC5444847.

23. Krolewiecki AJ, Lammie P, Jacobson J, Gabrielli AF, Levecke B, Socias E, et al. A public health response against Strongyloides stercoralis: time to look at soil-transmitted helminthiasis in full. PLoS Negl Trop Dis. 2013;7(5):e2165. doi: 10.1371/journal.pntd.0002165. PubMed PMID: 23675541; PubMed Central PMCID: PMCPMC3649958.

24. Lawrence G, Leafasia J, Sheridan J, Hills S, Wate J, Wate C, et al. Control of scabies, skin sores and haematuria in children in the Solomon Islands: another role for ivermectin. Bull World Health Organ. 2005;83(1):34-42. doi: /S0042-96862005000100012. PubMed PMID: 15682247; PubMed Central PMCID: PMCPMC2623469.

25. Masud H, Qureshi TQ, Dukley M. Effects of Ivermectin with and without doxycycline on clinical symptoms of onchocerciasis. J Coll Physicians Surg Pak. 2009;19(1):34-8. doi: 01.2009/JCPSP.3438. PubMed PMID: 19149978.

26. Osei-Atweneboana MY, Eng JK, Boakye DA, Gyapong JO, Prichard RK. Prevalence and intensity of Onchocerca volvulus infection and efficacy of ivermectin in endemic communities in Ghana: a two-phase epidemiological study. Lancet. 2007;369(9578):2021-9. doi: 10.1016/S0140-6736(07)60942-8. PubMed PMID: 17574093.

27. Osei-Atweneboana MY, Awadzi K, Attah SK, Boakye DA, Gyapong JO, Prichard RK. Phenotypic evidence of emerging ivermectin resistance in Onchocerca volvulus. PLoS Negl Trop Dis. 2011;5(3):e998. doi: 10.1371/journal.pntd.0000998. PubMed PMID: 21468315; PubMed Central PMCID: PMCPMC3066159.

28. Ottesen EA, Hooper PJ, Bradley M, Biswas G. The global programme to eliminate lymphatic filariasis: health impact after 8 years. PLoS Negl Trop Dis. 2008;2(10):e317. doi: 10.1371/journal.pntd.0000317. PubMed PMID: 18841205; PubMed Central PMCID: PMCPMC2556399.

29. Pacque MC, Dukuly Z, Greene BM, Munoz B, Keyvan-Larijani E, Williams PN, et al. Community-based treatment of onchocerciasis with ivermectin: acceptability and early adverse reactions. Bull World Health Organ. 1989;67(6):721-30. PubMed PMID: 2633887; PubMed Central PMCID: PMCPMC2491319.

30. Potasman I, Feiner M, Arad E, Friedman Z. Cutaneous larva migrans, sacroileitis, and optic neuritis caused by an unidentified organism acquired in Thailand. J Travel Med. 1998;5(4):223-5. PubMed PMID: 9876201.

31. Richard-Lenoble D, Chandenier J, Duong TH. [Antiparasitic treatments in pregnant women and in children in 2003]. Med Trop (Mars). 2003;63(4-5):491-7. PubMed PMID: 14763305.

32. Richard-Lenoble D, Chandenier J, Gaxotte P. Ivermectin and filariasis. Fundam Clin Pharmacol. 2003;17(2):199-203. PubMed PMID: 12667230.

33. Rosenblatt JE. Antiparasitic agents. Mayo Clin Proc. 1992;67(3):276-87. PubMed PMID: 1545596.

34. Rosenblatt JE. Antiparasitic agents. Mayo Clin Proc. 1999;74(11):1161-75. doi: 10.4065/74.11.1161. PubMed PMID: 10560606.

35. Sheele JM, Byers PA, Sonenshine DE. Initial assessment of the ability of ivermectin to kill Ixodes scapularis and Dermacentor variabilis ticks feeding on humans. Wilderness Environ Med. 2013;24(1):48-52. doi: 10.1016/j.wem.2012.08.003. PubMed PMID: 23131756.

36. Soungalo T, Soumana D, Moussa S, Francois R. Ivermectin in onchocerciasis control in the forest zone of Cote d'Ivoire. Acta Trop. 1997;68(3):297-300. PubMed PMID: 9492914.

37. Stephenson I, Wiselka M. Drug treatment of tropical parasitic infections: recent achievements and developments. Drugs. 2000;60(5):985-95. PubMed PMID: 11129130.

38. Taylor MJ, Hoerauf A, Bockarie M. Lymphatic filariasis and onchocerciasis. Lancet. 2010;376(9747):1175-85. doi: 10.1016/S0140-6736(10)60586-7. PubMed PMID: 20739055.

39. Taylor MJ, Hoerauf A, Townson S, Slatko BE, Ward SA. Anti-Wolbachia drug discovery and development: safe macrofilaricides for onchocerciasis and lymphatic filariasis. Parasitology. 2014;141(1):119-27. doi: 10.1017/S0031182013001108. PubMed PMID: 23866958; PubMed Central PMCID: PMCPMC3884836.

40. Vaidhyanathan U. Review of ivermectin in scabies. J Cutan Med Surg. 2001;5(6):496-504. doi: 10.1177/120347540100500607. PubMed PMID: 11907859.

41. Walker GJ, Johnstone PW. Interventions for treating scabies. Cochrane Database Syst Rev. 2000;(3):CD000320. doi: 10.1002/14651858.CD000320. PubMed PMID: 10908470.

42. Whitworth JA, Downham MD, Lahai G, Maude GH. A community trial of ivermectin for onchocerciasis in Sierra Leone: compliance and parasitological profiles after three and a half years of intervention. Trop Med Int Health. 1996;1(1):52-8. PubMed PMID: 8673823.
